# *Rhodotorula mucilaginosa* DM2025 ameliorates obesity through cholesterol binding and the gut mycobiome–BAT PPARγ/UCP1 axis

**DOI:** 10.1080/19490976.2026.2760059

**Published:** 2026-11-10

**Authors:** Yikang Jing, Yinghan Xiang, Oumarou Chaibou Zafir, Qiqi Sun, Bingwang Chen, Xinran Cao, Jie Hou, Huajun Li

**Affiliations:** a Department of Pathogen Biology and Microecology, College of Basic Medical Sciences, Dalian Medical University, Dalian, PR China; b Department of Blood Transfusion, Xi'an Daxing Hospital, Xi'an, PR China; c The First Affiliated Hospital, Dalian Medical University, Dalian, PR China; d Department of Biotechnology, College of Basic Medical Sciences, Dalian Medical University, Dalian, PR China

**Keywords:** *Rhodotorula mucilaginosa*, obesity, gut mycobiome, brown adipose tissue, thermogenesis

## Abstract

Obesity is a major global health challenge, necessitating safe and effective therapies. Although probiotics are promising for obesity management, research has focused on bacteria, leaving non-*Saccharomyces* yeasts underexplored. Here, we investigated the anti‑obesity mechanisms of *Rhodotorula mucilaginosa* DM2025, isolated from the healthy murine gut. To test whether benefits depend on viable cells, high‑fat diet‑fed mice received either live yeast or its cell‑free supernatant, followed by integrated physiological, genomic, and metabolomic analyzes. Live yeast, but not supernatant, significantly reduced body weight gain, improved serum lipids, and was associated with activation of brown adipose tissue thermogenesis (upregulated PPARγ, PGC1α, UCP1). Though the supernatant lowered total cholesterol, it failed to affect weight or thermogenic genes, indicating cholesterol‑lowering and weight reduction are distinct processes. Live cells were enriched in propionate, indole, and carotenoid‑related metabolites, with genomic support for their biosynthesis. Live yeast reshaped the gut mycobiome, promoting *Basidiomycota*/*Rhodotorula* dominance; this fungal restructuring was absent in HFD controls. These findings suggest that *R. mucilaginosa* DM2025 anti‑obesity efficacy relies on the continuous metabolic activity of live cells, producing thermogenesis‑associated metabolites that may contribute to BAT activation. Additionally, physical cholesterol binding to the yeast cell surface provides a complementary cholesterol‑reduction mechanism. Thus, *R. mucilaginosa* DM2025 represents a promising live probiotic candidate for obesity intervention.

## Graphical abstract

**Figure:**
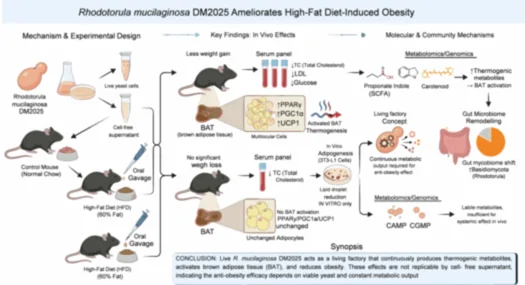

## Introduction

Obesity has become a global pandemic and is a major risk factor for numerous metabolic diseases, including diabetes mellitus, dyslipidemia, and cardiovascular disease.^1-3^ Its pathogenesis is multifactorial, involving dysregulated energy homeostasis, chronic low‑grade inflammation, and adipose tissue dysfunction. Among these mechanisms, brown adipose tissue (BAT) plays a pivotal role in energy expenditure through uncoupling protein 1 (UCP1)-mediated non-shivering thermogenesis.^4^ ^,^ ^5^ Therefore, strategies that activate BAT have attracted considerable attention as promising therapeutic approaches for obesity and related metabolic disorders.^6^

Increasing evidence indicates that the gut mycobiome is an important regulator of host metabolism and energy homeostasis.^7^ Although the contribution of the bacterial microbiome to metabolic diseases has been extensively investigated, the functional role of the gut mycobiome in obesity remains poorly understood. A deeper understanding of host–fungal interactions may therefore provide new insights into the development of microbiome-based therapeutic strategies for metabolic diseases.

Probiotic supplementation has emerged as a potential strategy for ameliorating obesity and associated metabolic disorders.^8^ However, probiotic research has focused predominantly on bacterial species, particularly *Lactobacillus* and *Bifidobacterium*, whereas the therapeutic potential of probiotic yeasts remains relatively underexplored. Accumulating evidence suggests that *Saccharomyces boulardii*, *Kluyveromyces marxianus*, and other yeast strains exert beneficial effects on host metabolism, indicating the latent value of yeast probiotics.^9^ ^,^ ^10^ ​​​​​

*Rhodotorula mucilaginosa* is a pigmented yeast widely distributed in natural environments and mammalian hosts. It produces various bioactive carotenoids, including torularhodin, *β*-carotene, *γ*-carotene, and torulene, that exhibit strong antioxidant activity.^11^ ^,^ ^12^ In addition, *R. mucilaginosa* has been evaluated as a feed additive to improve digestibility and gut health in animals.^13^ Despite these promising characteristics, its direct role in regulating host metabolism and obesity remains poorly understood. In our previous study, amphotericin B-mediated depletion of the gut mycobiome identified *Rhodotorula* as a keystone taxon associated with improved lipid metabolism and BAT thermogenesis.^14^ In the same study, we isolated *R. mucilaginosa* DM2025 from healthy murine gut and demonstrated its cholesterol-lowering capacity *in vitro*. These observations suggested a potential role for *Rhodotorula* in maintaining metabolic homeostasis. However, correlative evidence from fungal depletion and *in vitro* assays does not establish a causal relationship *in vivo*. Whether *R. mucilaginosa*, when administered as a live probiotic, directly alleviates obesity, and whether its metabolic effects depend on viable cells or their secreted metabolites, remains unknown. Moreover, a recent gnotobiotic study reported that early-life colonization with live *R. mucilaginosa* promoted adiposity in mice,^15^ emphasizing that the metabolic effects of this yeast may be highly dependent on the host context and mode of administration.

To address these questions, we evaluated the anti-obesity effects of live *R. mucilaginosa* DM2025 versus its cell‑free supernatant in HFD-fed mice, and further integrated genomic and metabolomic approaches to dissect the underlying mechanisms, focusing on cholesterol binding, gut mycobiome remodeling, and activation of the BAT PPARγ/UCP1 thermogenic axis.

## Materials and methods

### Strain culture and supernatant preparation

*R. mucilaginosa* DM2025, originally isolated from the intestine of a healthy mouse, was grown in YM liquid medium (1% yeast extract, 2% peptone, 2% glucose) at 25 °C with shaking at 300 rpm for 24 h. The culture was subsequently centrifuged at 3500 × g for 10 min at 4 °C to separate the cell pellet from the culture supernatant. The cell pellet was washed twice with sterile saline and resuspended to a final concentration of 1 × 10^8^ CFU/mL. The culture supernatant of *R. mucilaginosa* DM2025 was filter-sterilized through a 0.22 μm membrane, aliquoted, and stored at −80 °C until further use.

### *In vitro* assessment of probiotic properties and biosafety

#### Survival ability under simulated gastrointestinal conditions

Simulated gastric fluid (SGF) and simulated intestinal fluid (SIF) were prepared according to the Chinese Pharmacopoeia. For SGF: 10 g of pepsin was dissolved in 1 L of distilled water, and the pH was adjusted to 3.0 using dilute hydrochloric acid. For SIF, 6.8 g of potassium dihydrogen phosphate (KH₂PO₄) was dissolved in 1 L of distilled water, and the pH was adjusted to 6.8 with 0.1 mol/L sodium hydroxide solution. Subsequently, 10 g of pancreatin was added and completely dissolved. Both SGF and SIF were sterilized by filtration through 0.22 μm membranes and dispensed into 10 mL aliquots.

To evaluate gastrointestinal tolerance, *R. mucilaginosa* DM2025 was cultured for 24 h to obtain actively growing cells. The culture was centrifuged at 3500 × g for 10 min at 4 °C, and the resulting cell pellet was resuspended in sterile saline to a final concentration of 1 × 10^8^ CFU/mL. The yeast suspension was inoculated into the prepared SGF and SIF at 10% (v/v) and incubated at 37 °C. Samples were collected at 0, 3, and 6 h, serially diluted, and plated for colony counting. The viable count at 0 h served as the control for calculating survival rates. All experiments were performed in triplicate.

#### Bile salt tolerance

YM liquid medium and a 1% (w/v) bile salt solution prepared in sterile saline were autoclaved separately and subsequently mixed to obtain YM‑bile salt medium containing final bile salt concentrations of 0.1%, 0.3% (w/v). A 5% (v/v) inoculum of *R. mucilaginosa* DM2025 pre‑cultured for 24 h, was inoculated into each bile salt- containing medium and incubated at 37 °C. Samples were collected at 0 and 6 h, serially diluted, and plated for viable colony counting. The viable count at 0 h was used as the baseline for calculating the survival rate. All experiments were performed in triplicate.

#### Auto‑aggregation and hydrophobicity assays

Auto‑aggregation and hydrophobicity were determined according to the method described by Bonatsou et al.^16^

Auto-aggregation assay: an overnight culture of *R. mucilaginosa* DM2025 was centrifuged at 3500 × g for 10 min at 4 °C. The supernatant was discarded, and the cells were washed twice with sterile physiological saline. The cell pellet was resuspended to an optical density of 0.5 ± 0.02 at 620 nm (A₀). The cell suspension was vortexed for 15 s to ensure uniform dispersion and then incubated at 37 °C. At 0.5, 1, 1.5, 2, 2.5, 3, 6, 9, and 12 h, aliquots were carefully collected from the upper phase, and the absorbance at 620 nm was measured (A₁). Auto‑aggregation (%) was calculated using the following equation: Auto‑aggregation (%) = (1—A₁/A₀) × 100%.

Hydrophobicity assay: an overnight culture of *R. mucilaginosa* DM2025 was centrifuged at 3500 × g for 5 min at 4 °C. The cells were washed twice with PBS (pH 7.0) and resuspended to an OD620 of 0.5 ± 0.02 (A₀). A 3 mL aliquot of the cell suspension was mixed with 1 mL of xylene and vortexed vigorously for 2 min. After standing for 30 min to allow phase separation, the aqueous phase was carefully collected, and its absorbance at 620 nm was measured (A₁). All experiments were performed in triplicate. Hydrophobicity (%) was calculated using the same formula as above.

#### Hemolytic activity assay

An overnight culture of *R. mucilaginosa* DM2025 was spotted onto Columbia blood agar plates. After standing for 10 min, the plates were incubated at 30 °C for 48 h. Hemolytic activity was assessed by examining the presence or absence of a clear hemolytic zone surrounding the colonies, as previously described.^17^

#### Cholesterol removal assay

A cholesterol stock solution (10 mg/mL in anhydrous ethanol) was added to sterilized YM medium at 5% to prepare cholesterol-supplemented YM (YM-CHOL). *R. mucilaginosa* DM2025 was adjusted to 1 × 10^8^ CFU/mL and inoculated into 10 mL YM-CHOL at 1%, followed by incubation at 27 °C, 300 rpm for 24 h. After centrifugation (3500 × g, 10 min, 4 °C), the supernatant was collected and cholesterol concentration was measured using a total cholesterol assay kit (microplate method) according to the manufacturer’s instructions. Uninoculated YM-CHOL (blank control, B) and inoculated YM without cholesterol (yeast-only control, S₀) were processed in parallel. The cholesterol removal rate was calculated as: [B − (S − S₀)]/B × 100%, where S is the cholesterol concentration in the supernatant of the cholesterol-supplemented culture. All experiments were performed in triplicate.

#### Scanning electron microscopy (SEM) of *R. mucilaginosa* DM2025 cells and cholesterol adhesion

*R. mucilaginosa* DM2025 was activated by culturing for 24 h, after which a 2% (v/v) inoculum was transferred into fresh YM liquid medium and incubated at 30 °C with shaking for 24 h. The culture was centrifuged at 3500 × g for 10 min at 4 °C, and the supernatant was discarded. The fungal cell pellet was washed 2–3 times with PBS (pH 7.0). The washed cells were resuspended in an electron microscopy fixation solution and incubated at room temperature in the dark for 30 min for initial fixation, followed by overnight fixation at 4 °C. The next day, the fixed cells were centrifuged again at 3500 × g for 10 min, after which the supernatant was discarded. The samples were subsequently subjected to critical‑point drying, gold sputter‑coating under vacuum, and observed using a field‑emission scanning electron microscope.

For cholesterol adhesion observation, the same fungal cell preparation was incubated with a cholesterol solution (0.5 mg/mL) at 30 °C for 24 h, washed three times with PBS to remove unbound cholesterol, and then processed for SEM as described above.

## 3T3‑L1 preadipocyte differentiation and Oil Red O staining

3T3‑L1 preadipocytes were purchased from Suzhou Haixing Biotechnology Co., Ltd. Cells were cultured in complete 3T3‑L1 medium at 37 °C in a humidified atmosphere containing 5% CO_2_. Upon reaching 80% confluence, the cells were seeded into 6-well plates and induced to differentiate using 3T3-L1 adipogenic differentiation medium. After 3 d, the differentiation medium was replaced with maintenance medium, and this induction-maintenance cycle was repeated until day 12 to achieve complete adipocyte differentiation. Fully differentiated adipocytes were subsequently treated with *R. mucilaginosa* DM2025 cell-free supernatant at final concentrations of 5% and 10% for 48 h, after which they were subjected to further analyzes.

To evaluate intracellular lipid accumulation, Oil Red O staining was performed following treatment. Cells were fixed with 4% formaldehyde for 45 min and stained with Oil Red O solution for 30 min at room temperature. After staining, the cells were washed twice with PBS, observed under a microscope, and photographed. Quantitative analysis of lipid accumulation was performed using ImageJ software by measuring the Oil Red O-positive area.

### Whole-genome sequencing and identification of strain DM2025

A 24‑h culture of *R. mucilaginosa* DM2025 was collected and centrifuged at 3500 × g for 10 min, washed twice with PBS, and subjected to genomic DNA extraction using a commercial DNA extraction kit. DNA concentration, purity, and integrity were assessed before sequencing. Whole‑genome sequencing was performed using a combination of Illumina NovaSeq and PacBio SMRT platforms. Raw reads were quality‑filtered and assembled de novo using MEGAHIT (version 1.1.2), with gaps closed using GapCloser (version 1.12). Assembly completeness was evaluated using BUSCO (version 5.4.5) against the fungal ortholog database.^18^

Gene prediction was performed using MAKER2 (version 2.32), integrating the ab initio predictors Augustus (version 3.5.0) and SNAP. tRNA and rRNA genes were predicted using tRNA scan‑SE (version 2.0.12) and Barrnap (version 0.9), respectively. Functional annotation of predicted protein‑coding genes was performed by BLASTP searches against the NCBI Non‑Redundant Protein Database (NR), the Clusters of Orthologous Groups (COG) database for KOG classification, and the Kyoto Encyclopedia of Genes and Genomes (KEGG) database for pathway mapping.^19^ Carbohydrate‑active enzymes were annotated using the CAZy database.^20^ For safety assessment, antimicrobial resistance genes were identified using the Comprehensive Antibiotic Resistance Database (CARD), whereas putative virulence-associated genes were predicted using the Virulence Factors Database (DFVF) and the Pathogen–Host Interaction Database (PHI‑base).^21-23^

### Untargeted metabolomics analysis of *R. mucilaginosa* DM2025 and its cell-free supernatant

A 24‑h culture of *R. mucilaginosa* DM2025 was centrifuged at 3500 × g for 10 min to separate the cell pellet from the culture supernatant. The supernatant was filtered through a 0.22 μm membrane to obtain a cell-free supernatant, while the cell pellet was washed twice with PBS and collected. Untargeted metabolomics analysis was performed by Shanghai Majorbio Biotech Co., Ltd.

Raw metabolic data were processed using Progenesis QI software (Waters Corporation, Milford, USA) for peak detection, feature extraction, alignment, and peak integration. Multivariate statistical analyzes, including principal component analysis (PCA) and orthogonal partial least‑squares discriminant analysis (OPLS‑DA), were conducted using the ropls package (version 1.6.2) in R. Metabolite annotation was conducted against the Human Metabolome Database (HMDB), METLIN, and the Kyoto Encyclopedia of Genes and Genomes (KEGG) database. Pathway enrichment analysis was performed using the hypergeometric test in Python.

### Animals and housing

Male C57BL/6J mice (5–6 weeks old, body weight approximately 20 ± 2 g) were obtained from Liaoning Changsheng Biotechnology Co., Ltd, with the laboratory animal use permit number SYXK(Liao)2020-0001. All mice were housed in an SPF facility under controlled environmental conditions (temperature 22–25 °C, relative humidity 50%–70%, 12 h light/dark cycle) with free access to food and water. All animal procedures were approved by the Animal Ethics Committee of Dalian Medical University (approval No. XL251114609).

### Evaluation of the anti‑obesity effect of *R. mucilaginosa* DM2025

To evaluate the anti‑obesity potential of *R. mucilaginosa* DM2025, 21 male C57BL/6J mice (5–6 weeks old) were acclimated for 1 week and then randomly assigned to three experimental groups (*n* = 7 per group): (1) normal chow diet (NC), (2) high‑fat diet (HFD), and (3) HFD supplemented with *R. mucilaginosa* DM2025 (HFD + RM). The HFD and HFD + RM groups were fed a 60% high‑fat diet (5.24 kcal/g; Jiangsu Medison Biomedical Co., Ltd.), while the NC group received a standard maintenance diet (3.79 kcal/g). The HFD + RM group received a daily oral gavage of 0.2 mL *R. mucilaginosa* DM2025 suspension (1 × 10^8^ CFU/mL) for 6 weeks, whereas the NC and HFD groups received an equal volume of normal saline. Body weight and food intake were recorded weekly throughout the experimental period. At the end of the intervention, mice were fasted overnight and euthanized by CO₂ asphyxiation. Blood, epididymal fat, brown adipose tissue (BAT), and cecal contents were collected for subsequent analyzes.

### Serum biochemical analysis

Serum total cholesterol (TC) was measured using the glycerol cholesterol oxidase‑peroxidase (GOD‑PAP) method. TC concentration was calculated as: TC concentration = (sample optical density [OD] − blank OD)/(calibrator OD − blank OD) × calibrator concentration. Low‑density lipoprotein cholesterol (LDL‑C) was determined by a visible‑light homogeneous assay. Both TC and LDL‑C were quantified using commercially available kits (Nanjing Jiancheng Bioengineering Institute, China) according to the manufacturer's instructions.

### Histopathological analysis

Brown adipose tissue (BAT) and epididymal white adipose tissue (eWAT) were collected from mice, and immediately fixed in 4% paraformaldehyde solution at room temperature. The tissues were then embedded in paraffin, sectioned at a thickness of 5 μm, and stained with hematoxylin and eosin (HE). Images were captured under a light microscope (Nikon Instruments Co., Ltd., Japan) at magnifications of 40×, 100×, and 400×. Histopathological evaluation of adipose tissues focused on the degree of adipocyte hypertrophy. For quantitative analysis, randomly selected adipocytes were measured for cell area at 400× magnification using ImageJ software.

### RNA isolation and real-time quantitative PCR

Total RNA was extracted from BAT using the Seven‑Fast Total RNA Extraction Kit (Beijing Seven Innovation Biotechnology Co., Ltd., Beijing, China). RNA concentration and purity were determined using a NanoDrop spectrophotometer. Subsequently, cDNA was synthesized using a reverse transcription kit (Genstar, Beijing, China). The mRNA expression levels of PPARγ, PGC1α, and UCP1 were quantified using the LineGene 9600 Plus system with SYBR Green Chemistry (Genstar, Beijing, China). The specific primers used are listed in Table S1 (synthesized by Beijing Tsingke Biotech Co., Ltd., Beijing, China). Each qPCR reaction mixture contained 10 μL of 2× SYBR Master Mix, 100 ng of template cDNA, 0.5 μL of each primer at 10 μM concentration, and nuclease‑free water to a final volume of 20 μL. The amplification program included an initial denaturation at 94 °C for 30 s, followed by 40 cycles of denaturation at 95 °C for 10 s and annealing at 60 °C for 30 s. β‑Actin was used as the internal reference gene, and relative expression levels were calculated using the 2^-^ΔΔCt method.

### Direct comparison between live *R. mucilaginosa* DM2025 and cell‑free supernatant

To determine whether the anti‑obesity effects of *R. mucilaginosa* DM2025 are mediated by live cells or by secreted metabolites, an independent cohort of 32 male C57BL/6J mice was acclimated for 1 week and randomly assigned to four experimental groups (*n* = 8 per group): (1) NC, (2) HFD, (3) HFD + RM (live *R. mucilaginosa* DM2025 1 × 10^8^ CFU/mL), and (4) HFD + SUP (cell‑free supernatant, 0.2 mL per mouse). All treatments were administered via daily oral gavage for 6 weeks. The HFD + SUP group received the sterile supernatant at the corresponding concentration. Body weight and food intake were recorded weekly throughout the intervention period.

At the end of the experiment, mice were euthanized as described above. Blood, epididymal white adipose tissue (eWAT), and BAT were collected for subsequent analyzes. Serum TC and LDL-C concentrations were determined as described in the Serum biochemical analysis section, and the mRNA expression levels of PPARγ, PGC1α, and UCP1 in BAT were analyzed by RT-qPCR following the protocol described in Section QPCR, using the primer sequences listed in Table S1.

### Metagenomic sequencing of cecal contents

To evaluate the effects of live *R. mucilaginosa* DM2025 on the gut microbial community structure, cecal contents (0.1 g) collected from mice in Experiment 1 were used for DNA extraction following the manufacturer's protocol of a stool DNA isolation kit. DNA concentration, purity, and integrity were assessed by 1% agarose gel electrophoresis. DNA was fragmented using a Covaris M220, and fragments of approximately 400 bp were selected for paired‑end library construction using the NEXTFLEX Rapid DNA‑Seq kit. Metagenomic sequencing was performed on an Illumina MiSeq PE300 platform at Shanghai Majorbio Bio‑Pharm Technology Co., Ltd. Raw reads were aligned to the host genome using BWA (version 0.7.17) to remove contaminating host sequences. Clean reads were assembled using MEGAHIT (version 1.1.2). The amino acid sequences of the non‑redundant gene set were aligned against the KEGG database using Diamond (version 2.0.13).

### Statistical analysis

Data were analyzed using GraphPad Prism version 9.5.1 (GraphPad Software, Inc., La Jolla, CA, USA). All results are presented as mean ± standard deviation (SD). One-way ANOVA followed by Tukey's multiple comparisons test was used for group-wise comparisons of physiological and molecular data. For metagenomic and metabolomic analyzes, the Benjamini–Hochberg procedure was applied to control the false discovery rate. Normality and homogeneity of variances were tested before performing ANOVA. A *P*‑value < 0.05 was considered statistically significant.

## Results

### *R. mucilaginosa* DM2025 exhibits cholesterol binding, probiotic properties, and safety

Scanning electron microscopy revealed that *R. mucilaginosa* cells exhibited an oval to spherical shape with a smooth cell surface (Figure 1A). On YM agar, colonies initially appeared pale pink and turned orange–red upon extended incubation (Figure 1D); the strain was Gram-positive with monopolar budding (Figure 1E). Growth curve analysis indicated entry into exponential phase at 6 h and stationary phase at 24–30 h, reaching 2.2 × 10^12^ CFU/mL (Figure 1F).

**Figure 1.**
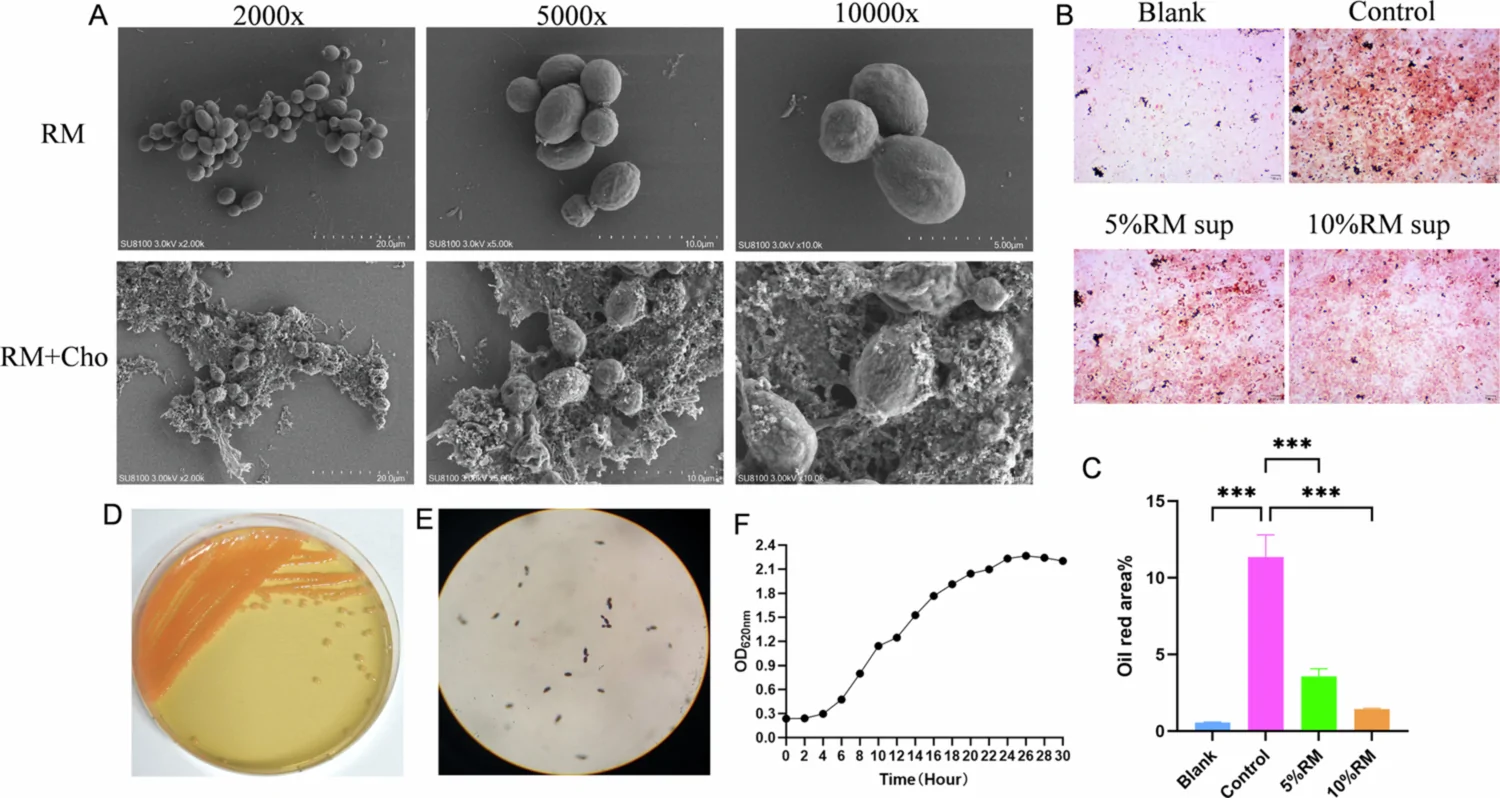
Characterization of *R. mucilaginosa* DM2025 and its effect on adipocyte differentiation. (A) SEM images of *R. mucilaginosa* DM2025 cells (top) and cholesterol adhesion (bottom). (B) Oil Red O staining of 3T3-L1 adipocytes. (C) Quantification of Oil Red O‑positive area using ImageJ software. (D) Colony morphology on YM agar after 48 h at 25 °C. (E) Light microscopy image after Gram staining. (F) Growth curve in YM liquid medium. (*P < 0.05, **P < 0.01, ***P < 0.001).

Our previous study demonstrated that *R. mucilaginosa* DM2025 possesses *in vitro* cholesterol-lowering activity.^14^ To further quantify this effect, we performed a cholesterol removal assay using a total cholesterol assay kit. *R. mucilaginosa* DM2025 cells removed 44.35% ± 4.35% of cholesterol from YM-CHOL medium after 24 h. To visualize the physical interaction between yeast cells and cholesterol, we further examined the cell surface using SEM. Following cholesterol incubation, numerous cholesterol crystals were observed adhering to the cell surface (Figure 1A), indicating that physical binding to cholesterol may serve as one of the mechanisms contributing to its cholesterol-lowering effects.^24^

An effective probiotic must remain viable during gastrointestinal transit. Therefore, the tolerance of DM2025 to simulated gastric fluid (SGF, pH 3.0) and simulated intestinal fluid (SIF, pH 6.8) was evaluated. In SIF, the survival rate of DM2025 was 79.87% ± 1.08% after 3 h and 65.17% ± 2.36% after 6 h. In SGF, the survival rate was 59.57% ± 0.56% after 3 h and 38.83% ± 1.17% after 6 h (Table S2). The strain also exhibited excellent bile salt tolerance, maintaining survival rates exceeding 85% after 6 h of exposure to both 0.1% and 0.3% bile salt concentrations (Table S3). In addition, DM2025 exhibited progressive auto-aggregation, reaching 81.2% after 12 h (Fig. S1), while its cell surface hydrophobicity toward xylene was 25.1%, indicating moderate adhesive capacity that may facilitate intestinal colonization.^16^

To evaluate the biosafety of *R. mucilaginosa* DM2025 for *in vivo* application, we performed a comprehensive genomic safety assessment (Fig. S2). Interrogation of the genome against the Virulence Factors Database (DFVF) identified several homologs annotated as fungal virulence-associated genes; however, these matches were based solely on sequence similarity and do not represent functional equivalence (Fig. S2A). Critically, no acquired antimicrobial resistance genes were detected in the Comprehensive Antibiotic Resistance Database (CARD), confirming the absence of transferable resistance determinants (Fig. S2B). Furthermore, screening against the Pathogen–Host Interaction Database (PHI) revealed no definitive pathogen–host interaction-related virulence factors; the few hits identified were all fungal conserved housekeeping genes with no established role in pathogenicity (Fig. S2C). These genomic findings are corroborated by phenotypic safety assessments, the strain exhibited no hemolytic activity on blood agar (γ‑hemolysis). Together, these integrated genomic and phenotypic data establish that *R. mucilaginosa* DM2025 poses negligible risk of pathogenicity or horizontal gene transfer, supporting its suitability for *in vivo* probiotic intervention.

To determine whether soluble metabolites produced by DM2025 possess biological activity relevant to lipid metabolism, we treated differentiating 3T3‑L1 preadipocytes with 5% and 10% cell‑free supernatant during the differentiation process. Oil Red O staining revealed that supernatant treatment markedly reduced lipid droplet accumulation in a dose‑dependent manner, with the 10% supernatant showing a more pronounced inhibitory effect than the 5% concentration (Figure 1B). Quantitative analysis confirmed that both concentrations significantly decreased the Oil Red O‑positive area compared with the control group *(P* < 0.001), with a greater reduction observed in the 10% supernatant group compared with the 5% group (Figure 1C). This observation suggests that *R. mucilaginosa* DM2025 produces bioactive metabolites with anti‑adipogenic potential. We therefore next investigated whether these soluble factors are sufficient to recapitulate the anti‑obesity effects *in vivo*.

### *R. mucilaginosa* DM2025 attenuates HFD-induced obesity and activates the BAT PPARγ–UCP1 thermogenic axis

To evaluate the anti-obesity potential of *R. mucilaginosa* DM2025 *in vivo*, we orally administered *R. mucilaginosa* DM2025 to HFD-fed mice for 6 weeks. Body weight monitoring revealed that RM-treated mice exhibited significantly lower weight gain from week 4 onward compared with HFD controls (Figure 2A). At the end of the intervention, the final body weight increment in the HFD + RM group was significantly lower than that in the HFD group (Figure 2B). This weight reduction was accompanied by marked improvements in serum biochemical parameters. Compared with HFD controls, RM administration significantly decreased serum total cholesterol (TC), low-density lipoprotein cholesterol (LDL-C), and fasting blood glucose levels (Figure 2C–E).

**Figure 2.**
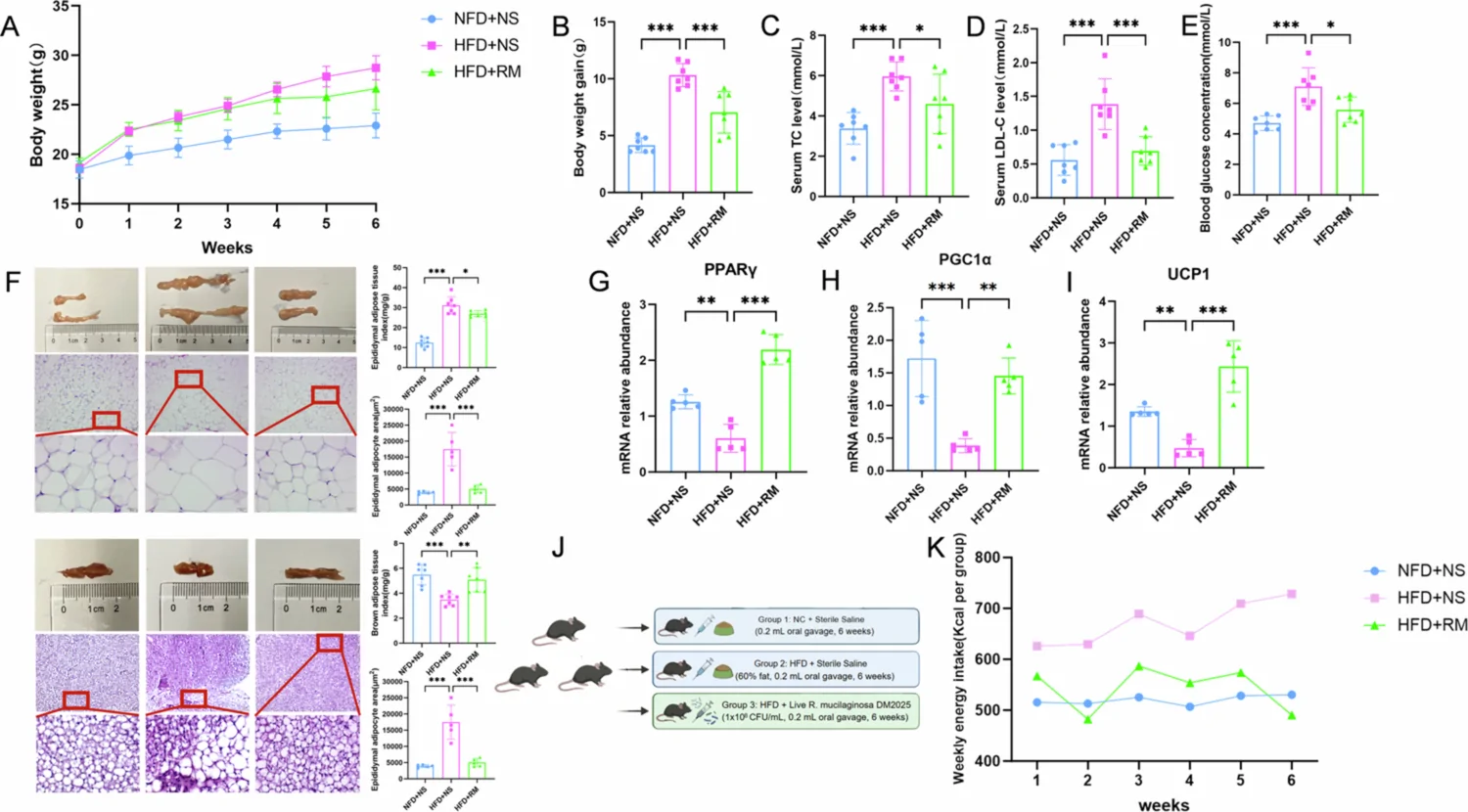
*R. mucilaginosa* DM2025 alleviates HFD-induced obesity and enhances BAT thermogenesis in mice. (A) Line graph of body weight gain in each group. (B) Final body weight increment. (C) Serum total cholesterol (TC). (D) Serum low-density lipoprotein cholesterol (LDL-C). (E) Blood glucose level. (F) H&E staining images of epididymal white adipose tissue and brown adipose tissue (BAT), with quantitative analysis of adipocyte area and fat indices. (G–I) qPCR analysis of PPARγ, PGC1α and UCP1 expression (*n* = 5). (J) Animal study design. (K) Weekly caloric intake (kcal/week) throughout the intervention. (*P < 0.05, **P < 0.01, ***P < 0.001).

Histological examination of epididymal white adipose tissue (eWAT) revealed that HFD feeding induced marked adipocyte hypertrophy. In contrast, RM treatment significantly reduced adipocyte size (*P* < 0.01; Figure 2F). Notably, brown adipose tissue (BAT) from HFD + RM mice displayed smaller multilocular lipid droplets and a denser cellular morphology compared with HFD-fed controls, consistent with enhanced BAT activity.^25^ ^,^ ^26^ The epididymal fat index was significantly decreased, whereas the BAT index was significantly increased in the RM group compared with HFD controls (Figure 2F).

To determine whether these morphological changes were associated with activation of the BAT thermogenic program, the expression of key thermogenesis-related genes was analyzed by RT-qPCR. Compared with the HFD group, RM treatment significantly increased the mRNA expression of PPARγ, PGC1α, and UCP1 in BAT (Figure 2G–I). Given that PPARγ is the master transcriptional regulator of BAT development, PGC1α drives mitochondrial biogenesis, and UCP1 directly mediates non-shivering thermogenesis,^4-6^ these coordinated upregulations are consistent with activation of the BAT thermogenic program. The experimental timeline is illustrated in Figure 2J. The HFD + RM group showed lower energy intake than the HFD group (Figure 2K).

### *R. mucilaginosa* DM2025 becomes the predominant fungal genus in the gut of HFD-fed mice

To evaluate the effect of *R. mucilaginosa* DM2025 on gut microbial community structure, metagenomic sequencing was performed on the cecal contents of mice. Principal coordinate analysis (PCoA) based on *β*-diversity revealed clear separation among groups, suggesting distinct microbial community structures (Figure 3A). Analysis of the bacterial community revealed that a HFD markedly altered the composition of gut bacteria at both the phylum and genus levels, whereas *R. mucilaginosa* DM2025 intervention did not result in an apparent restoration of the bacterial community, suggesting a limited impact of this strain on the gut bacteriome (Fig. S3). Notably, assessment of the microbial dysbiosis index (MDI) at the ASV level revealed that MDI was significantly higher in the HFD group compared with the NC group, whereas it was significantly reduced in the HFD + RM group compared with the HFD group (Fig. S4), indicating that although the bacterial community structure was not fully restored, the overall degree of microbial ecosystem dysbiosis was partially ameliorated by supplementation with *R. mucilaginosa* DM2025.

**Figure 3.**
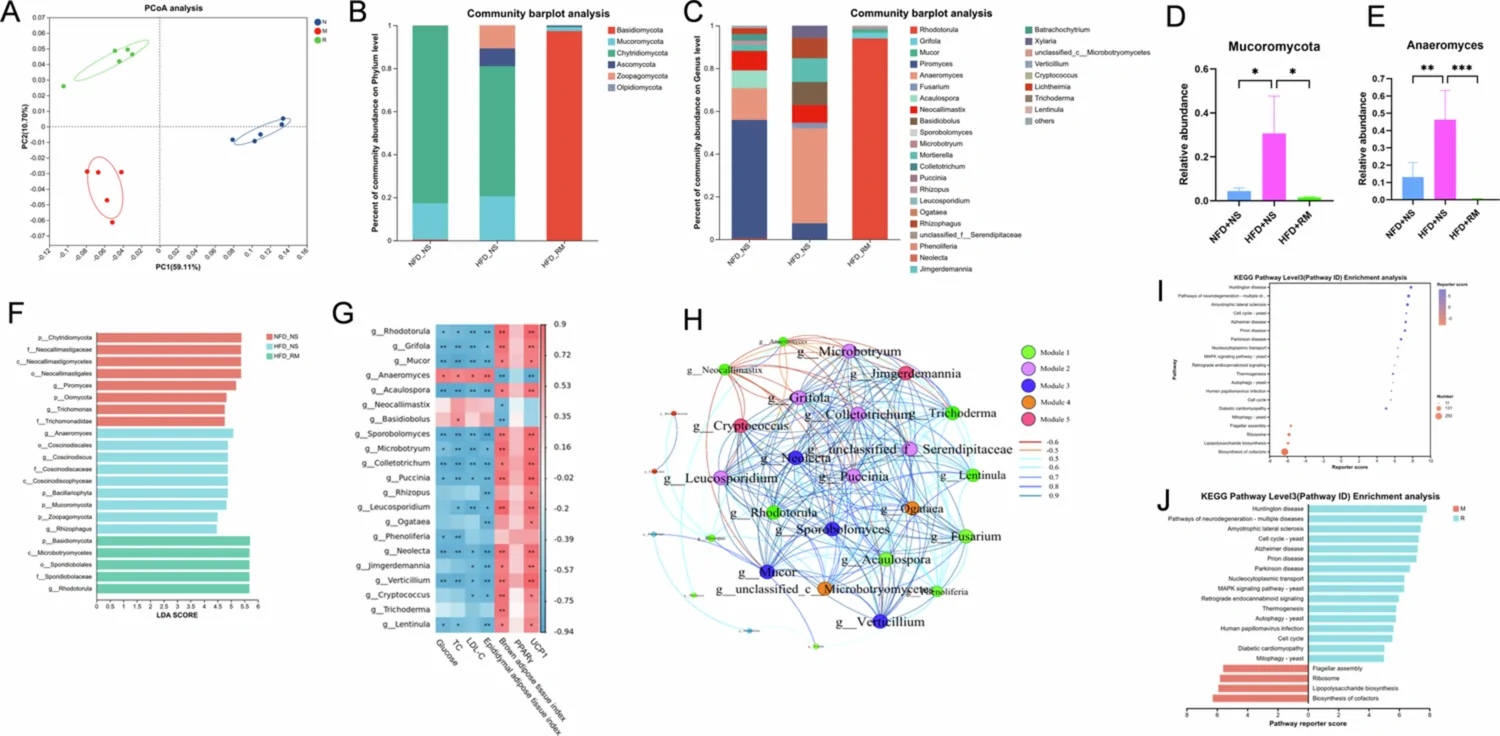
Impacts of *R. mucilaginosa* DM2025 on mouse gut microbiome. (A) Principal coordinate analysis (PCoA) of fungal communities among different groups. (B) Fungal community alterations at the phylum level. (C) Fungal community composition at the genus level. (D) Relative abundance of Mucoromycota. (E) Relative abundance of *Anaeromyces*. (F) LEfSe analysis of intestinal fungal communities. (G) Correlation analysis between intestinal fungal microbiota and biomarkers. (H) Fungal co-occurrence network analysis. (I) KEGG enrichment bubble plot. (J) Functional enrichment results of KEGG Level 3 pathways. All analyzes were performed with *n* = 5 per group. (*P < 0.05, **P < 0.01, ***P < 0.001).

In contrast, the fungal community exhibited pronounced differences at both phylum and genus levels. At the phylum level, the HFD + RM group was dominated by Basidiomycota (>90% relative abundance), while the NC and HFD groups were mainly composed of *Mucoromycota* and *Chytridiomycota* (Figure 3B). At the genus level, *Rhodotorula* overwhelmingly dominated the HFD + RM group, with only trace amounts detected in the NC group and none in the HFD group (Figure 3C). Compared with the HFD group, the HFD + RM group showed significantly reduced relative abundances of *Mucoromycota* and *Anaeromyces* (*P* < 0.001; Figure 3D–E). LEfSe analysis further identified Basidiomycota and *Rhodotorula* as signature taxa enriched in the HFD + RM group, whereas *Chytridiomycota, Mucoromycota*, and anaerobic genera such as *Anaeromyces* were associated with the NC and HFD groups (Figure 3F). LEfSe analysis of the bacterial community revealed no core taxa strongly associated with live yeast intervention (Fig. S3). Spearman correlation analysis between the HFD and HFD + RM groups revealed that genera including *Rhodotorula*, *Grifola*, and *Mucor* were significantly negatively correlated with glucose, TC, LDL‑C, and epididymal fat index, and positively correlated with BAT index and UCP1 expression (*P* < 0.05; Figure 3G). *Anaeromyces* exhibited opposite correlation patterns. Co‑occurrence network analysis identified *Rhodotorula* (module 1) and Grifola (module 2) as hub taxa with the highest core node indices (Figure 3H). PICRUSt2 functional prediction revealed that, compared with the HFD group, the HFD + RM group exhibited significant up‑regulation of KEGG pathways including thermogenesis, nucleocytoplasmic transport, and cell cycle; in contrast, the HFD group showed enrichment of pathways related to flagellar assembly, ribosome, and lipopolysaccharide biosynthesis (Figure 3I and J). Notably, the enrichment of the thermogenesis pathway in the live yeast group is consistent with the up‑regulation of PPARγ, PGC1α, and UCP1 expression in BAT observed in this study.

### *R. mucilaginosa* DM2025, but not its supernatant, reduces body weight and activates BAT thermogenesis

To directly compare the effects of live cells and secreted metabolites, we administered *R. mucilaginosa* DM2025 suspension and cell‑free supernatant to HFD‑fed mice via daily oral gavage for 6 weeks. Compared with the HFD group, the HFD + RM group showed significantly reduced body weight gain from week 2 onwards, whereas the HFD + SUP group exhibited no significant difference throughout the intervention (Figure 4A). At the end of the experiment, the final body weight increment in the HFD + RM group was significantly lower than that in the HFD group, while the HFD + SUP group showed no reduction (Figure 4B). Cumulative caloric intake was significantly reduced in the HFD + RM group compared with the HFD group, but not in the HFD + SUP group (Figure 4E). Serum total cholesterol (TC) was significantly decreased by both *R. mucilaginosa* DM2025 and supernatant, whereas LDL-C was reduced only by *R. mucilaginosa* DM2025 (Figure 4C and D). The BAT index (BAT weight/body weight) was significantly increased in the HFD + RM group compared with the HFD group, indicating enhanced BAT mass relative to body weight; no significant change was observed in the HFD + SUP group (Figure 4F). qPCR analysis of BAT revealed that mRNA expression levels of PPARγ, PGC1α, and UCP1 were significantly up‑regulated only in the HFD + RM group, with no significant changes in the HFD + SUP group compared with the HFD control (Figure 4G–I). The experimental design is summarized in Figure 4J.

**Figure 4.**
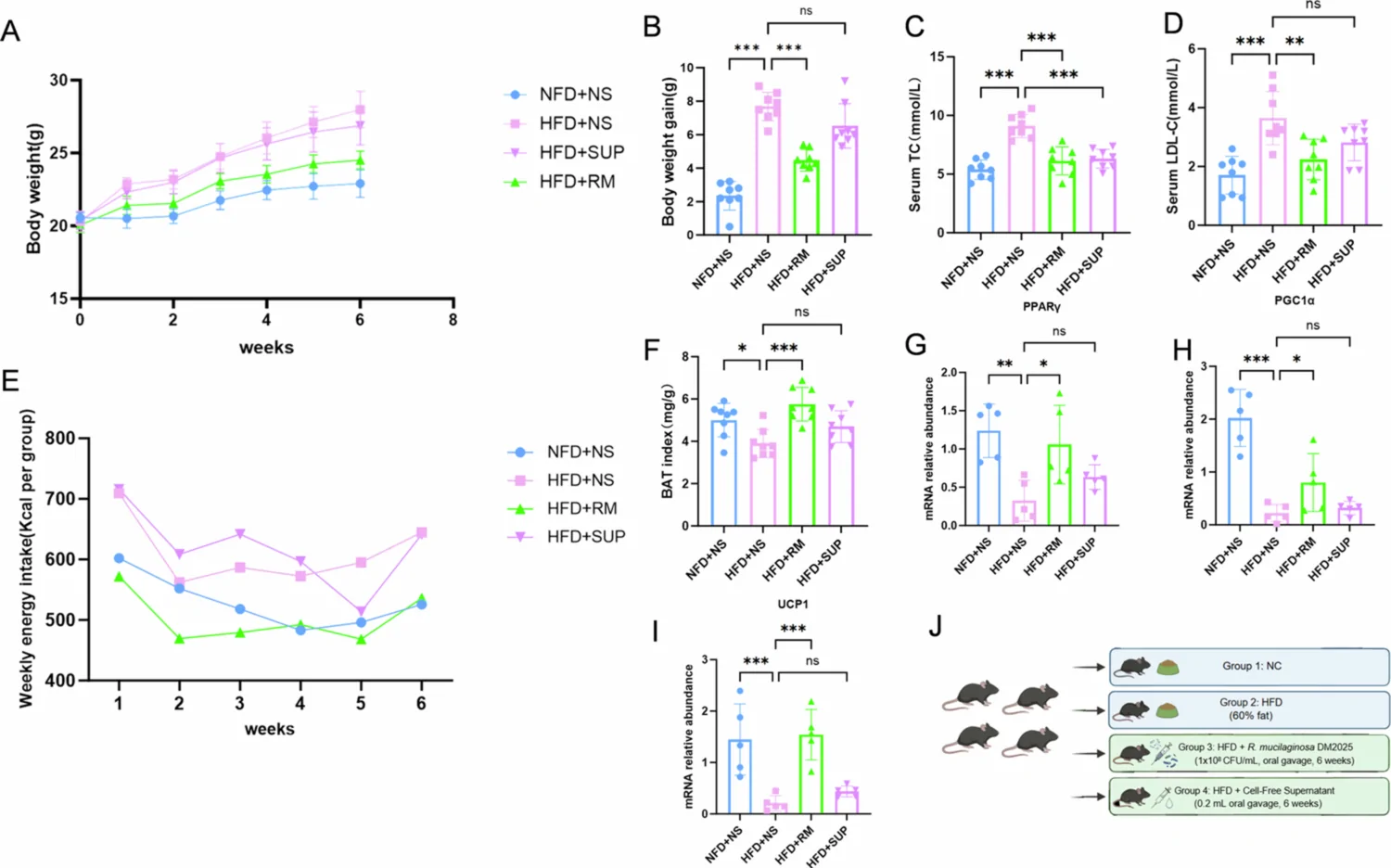
Effects of live *R. mucilaginosa* DM2025 and its supernatant (Sup) on high-fat diet (HFD)-induced obese mice. (A) Line graph of body weight gain in each group. (B) Final body weight increment. (C) Serum total cholesterol (TC). (D) Serum low-density lipoprotein cholesterol (LDL-C). (E) Weekly caloric intake (kcal/week) throughout the intervention. (F) Brown adipose tissue (BAT) index. (G–I) qPCR analysis of PPARγ, PGC1α and UCP1 expression (*n* = 5). (J) Animal study design. (*P < 0.05, **P < 0.01, ***P < 0.001).

### Genomic and metabolomic profiling of *R. mucilaginosa* DM2025

To explore the molecular basis underlying the BAT thermogenic activation associated with *R. mucilaginosa* DM2025, whole‑genome sequencing and untargeted metabolomics profiling were performed on the strain and its culture-derived fractions.

Whole‑genome sequencing and de novo assembly generated 19 contigs with a total genome size of approximately 21.4 Mb and an N50 value of 1 Mb. The assembled genome exhibited high completeness, with a BUSCO completeness score of 95.7% (Figure 5A). KEGG pathway classification revealed that the genome contains the largest number of genes involved in metabolism, particularly amino acid metabolism, carbohydrate metabolism, lipid metabolism, and terpenoid/polyketide metabolism (Figure 5B). KOG functional classification further showed significant enrichment of genes related to lipid transport and metabolism (category I) and secondary metabolite biosynthesis (category Q), whereas genes belonging to defense mechanisms (category V) and mobile elements (category X) were scarce or absent (Figure 5D). Annotation of carbohydrate‑active enzymes (CAZymes) revealed enrichment in glycoside hydrolases (GH, 66 genes) and glycosyltransferases (GT, 96 genes) (Figure 5C). These genomic features suggest that *R. mucilaginosa* DM2025 possesses genetic potential for active lipid metabolism and secondary metabolite production. Focused enrichment analysis of lipid metabolism-related genes confirmed that the *R. mucilaginosa* DM2025 genome is well equipped for lipid metabolism, spanning fatty acid and steroid biosynthesis, glycerophospholipid and sphingolipid metabolism, and lipase/acyltransferase activities (Supplementary Figs. S5 and S6).

**Figure 5.**
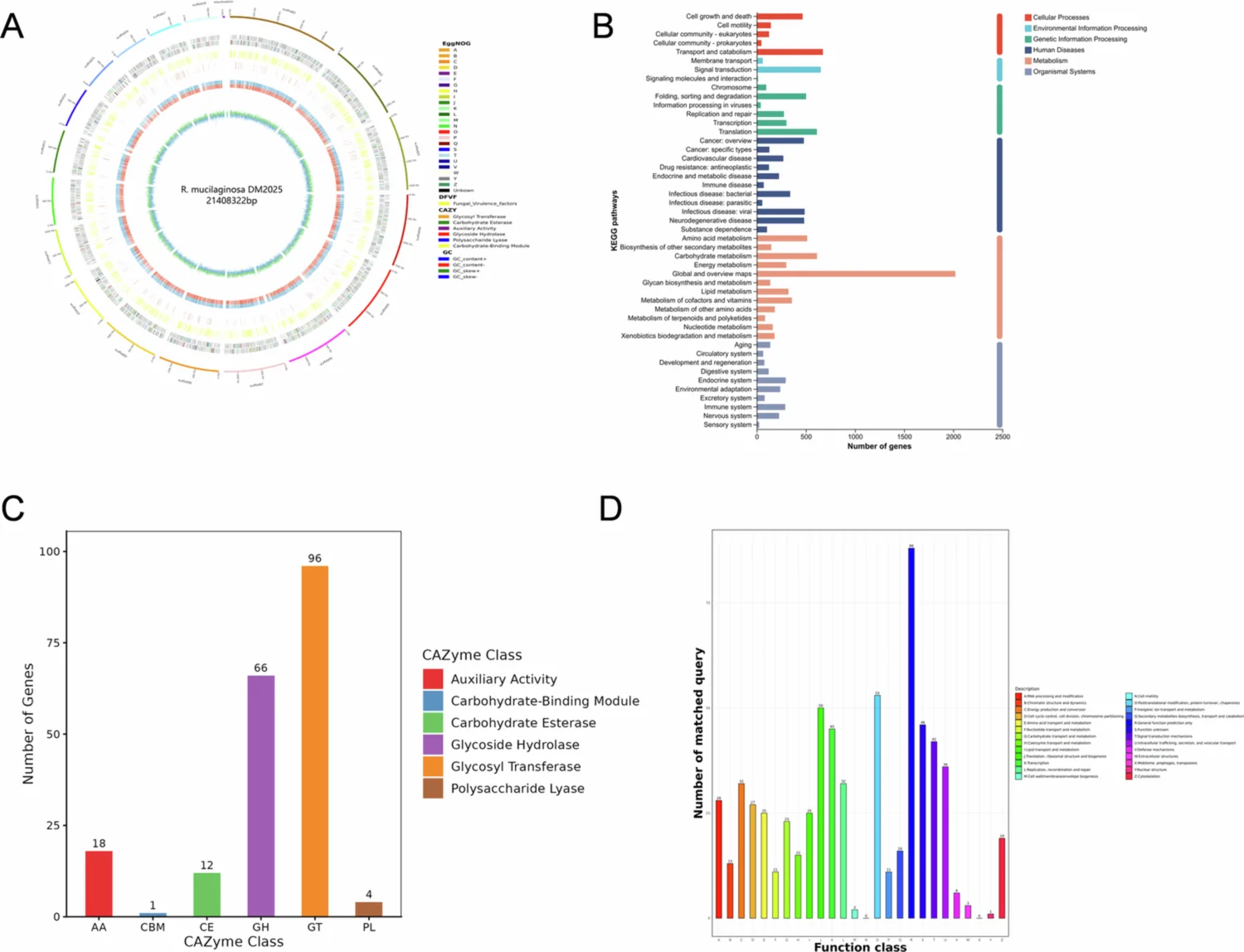
Whole-genome profiling. (A) Whole-genome circular map. (B) Functional annotation based on KEGG (Kyoto Encyclopedia of Genes and Genomes). (C) Functional annotation of carbohydrate-active enzymes (CAZyme). (D) Functional annotation based on KOG (Clusters of Orthologous Groups).

To identify metabolites that may mediate the observed metabolic benefits, we compared the metabolomic profiles of live cell pellets and cell‑free supernatant. Principal component analysis (PCA) showed clear separation between the two groups along the PC1 axis, with quality control samples clustering tightly, indicating good data reproducibility (Figure 6A). A total of 968 significantly differential metabolites were identified, of which 262 were up‑regulated and 706 down‑regulated in cell pellets compared with supernatant (Figure 6B). Notably, metabolites enriched in cell pellets included propionate, indole, anthranilic acid, L‑arginine, L‑valine, glutathione, acetyl‑L‑carnitine, sphinganine, and the carotenoid mactraxanthin (Figure 6C). By contrast, the supernatant exhibited higher abundance of cyclic nucleotides, including adenosine 3′,5′-cyclic monophosphate (cAMP), adenosine 2′,3′-cyclic phosphate and 3′,5′-cyclic GMP (cGMP) (Figure 6C). cAMP and cGMP are well-established second messengers that play critical roles in adipocyte differentiation, lipolysis, and thermogenic regulation.^27^ KEGG enrichment analysis of metabolites enriched in live cells revealed significant associations with thermogenesis, regulation of lipolysis in adipocytes, valine/leucine/isoleucine biosynthesis, and oxidative phosphorylation (Figure 6D), pathways closely linked to energy and lipid metabolism. Correspondingly, these pathway annotations align with the enrichment of propionate, indole, and carotenoid-related metabolites in live cell pellets, whose biosynthesis is supported by the strain's genomic repertoire.

**Figure 6.**
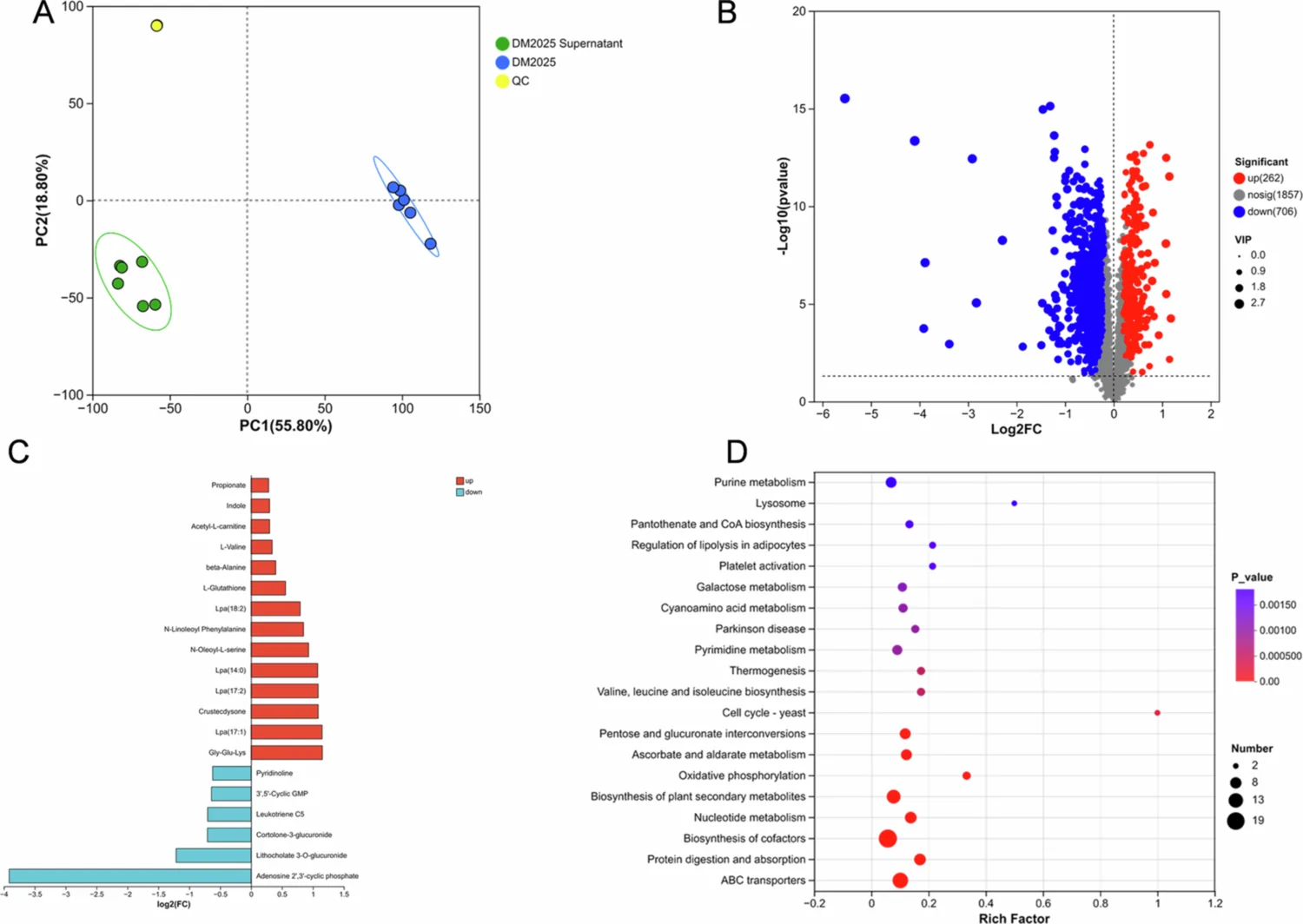
Untargeted metabolomics analysis of live cell pellets and supernatant. (A) Principal component analysis (PCA). (B) Volcano plot of differential metabolites from cell pellets and supernatants. (C) Significantly upregulated differential metabolites. (D) Functional annotation of differential metabolites based on KEGG.

## Discussion

Obesity has become a global public health challenge, and the gut microbiome has emerged as a key modifiable target for metabolic intervention.^28^ While probiotics have shown promise in obesity management, the vast majority of studies have centered on bacterial genera such as Lactobacillus and Bifidobacterium, leaving the therapeutic potential of non‑Saccharomyces yeasts largely uncharted.^8^ ^,^ ^29^ Notably, *Rhodotorula mucilaginosa*—a pigmented yeast widely distributed in nature and mammalian hosts—has yielded conflicting reports, with some studies suggesting beneficial metabolic effects and others linking early‑life colonization to increased adiposity.^15^ ^,^ ^30-32^ These contradictions raise a critical question: whether the metabolic impact of this yeast depends on its administration as live cells or as cell‑free metabolites, and whether its effects are context‑dependent. To address this, we directly compared live *R. mucilaginosa* DM2025 with its cell‑free supernatant in HFD‑fed mice using integrated physiological, genomic, and metabolomic analyzes.

This comparison revealed a striking dissociation: the supernatant reduced serum total cholesterol but failed to affect body weight or BAT thermogenesis, indicating that cholesterol lowering and weight loss represent distinct physiological processes. This concept is consistent with previous evidence showing that metabolic improvements and atherosclerosis resolution can occur independently of changes in circulating cholesterol concentrations.^33^ The cholesterol‑lowering effect of the supernatant may be partially attributed to soluble bioactive compounds present in the culture supernatant. Untargeted metabolomics revealed that the supernatant was enriched in cyclic nucleotides (e.g., cAMP and cGMP), which have been reported to inhibit hepatic HMG-CoA reductase (HMGCR) activity and suppress intestinal NPC1L1-mediated cholesterol absorption.^34-36^ These soluble metabolites may therefore reduce serum cholesterol through inhibition of HMGCR and/or suppression of intestinal cholesterol absorption. However, the supernatant lacks intact cells and cannot sustain the continuous production of these labile metabolites *in vivo*, which may explain why it reduced serum cholesterol without affecting body weight or BAT thermogenesis. In contrast to the supernatant, live yeast cells provide a sustained source of these metabolites and may additionally act through cell-dependent mechanisms. The EM observation of cholesterol crystals adhering to the yeast cell wall supports a cell-dependent mechanism by which *R. mucilaginosa* DM2025 can physically bind dietary cholesterol, consistent with the view that microbial cell surface binding contributes to cholesterol lowering.^24^ Similar cholesterol-binding capacity has been reported for other probiotic yeasts and bacteria, where cell surface components mediate cholesterol adhesion.^37^ ^,^ ^38^ Importantly, the structures observed by SEM were not identified on morphological grounds alone. In parallel, a quantitative enzymatic assay showed that live *R. mucilaginosa* DM2025 cells removed 44.35% ± 4.35% of cholesterol after 24 h, providing biochemical evidence of cholesterol binding. This combined morphological and biochemical approach is consistent with previous studies on cholesterol binding to microbial cell surfaces.^39^ ^,^ ^40^​​​​​​ This physical binding may reduce the amount of cholesterol available for intestinal absorption, providing a complementary mechanism for the cholesterol-lowering effect of live yeast. Although both live yeast and its supernatant reduced serum total cholesterol, only live yeast was associated with BAT thermogenesis activation and reduced body weight. These findings support the concept of live yeast as a “living factory”, in which intestinal colonization and continuous metabolism are irreplaceable by cell-free preparations.^41-43^ Metagenomic analysis further revealed that although *R. mucilaginosa* DM2025 administration did not fully restore the HFD‑disrupted bacterial community structure, it significantly reduced the microbial dysbiosis index (MDI). The reduction in MDI without significant restoration of bacterial community structure suggests that the improvement in microbial ecosystem homeostasis may be driven primarily by fungal compositional changes, rather than by a full reconstitution of the bacterial community.

The apparent discrepancy with Gutierrez et al.^15^ may be attributable to differences in host age, dietary context, intervention regimen, and co-colonizing microbiota, suggesting that the metabolic impact of *R. mucilaginosa* is highly context-dependent.^44^ These findings suggest that the metabolic impact of *R. mucilaginosa* may be context-dependent, and further studies are needed to identify the key host and microbial factors that determine whether this yeast promotes or ameliorates obesity.

The enrichment of *Rhodotorula* during continuous administration has been interpreted differently in current studies on *Rhodotorula* ecology in the gut. *Rhodotorula* is commonly detected in human stool samples, and several studies support its role as a resident or autochthonous member of the gut mycobiome.^45^ ^,^ ^46^ It has also been identified as a core member of the infant gut mycobiome.^15^ ^,^ ^47^ In a recent study of critically ill patients and healthy controls, *Rhodotorula* was one of the major fungal genera in healthy adults, and its abundance was higher in females than in males and depleted in sepsis and trauma patients.^48^ In gnotobiotic mice, *R. mucilaginosa* efficiently colonized the gut alongside *C. albicans*, with species-specific qPCR confirming true colonization rather than transient passage.^44^ However, its optimal growth temperature is below body temperature, its overgrowth is normally suppressed under physiological conditions, and it did not persist in mice two weeks after cessation of oral administration in at least one longitudinal study.^45^ ^,^ ^46^ These observations indicate that *Rhodotorula* is generally not a strict core member of the healthy adult gut mycobiome, and its persistence may depend on environmental factors. Consistent with this, our *in vitro* assays showed that *R. mucilaginosa* DM2025 survives simulated gastric and intestinal fluids, tolerates bile salts, and exhibits moderate auto-aggregation and hydrophobicity, indicating that it can survive gastrointestinal transit and may adhere to the intestinal mucosa to some extent. Together, these findings suggest that the enrichment of *Rhodotorula* observed during daily administration may reflect either transient passage with luminal interaction or partial colonization, and that host age, diet, sex, and co-colonizing microbiota may shape its persistence. The mechanistic basis for the differential efficacy between live yeast and its supernatant was further explored through integrated genomic and metabolomic analyzes. Whole‑genome sequencing showed that *R. mucilaginosa* DM2025 possesses a genomic repertoire enriched in genes involved in lipid metabolism, secondary metabolite biosynthesis, and carbohydrate‑active enzymes, particularly glycoside hydrolases and glycosyltransferases, supporting its capacity for active metabolic output. Untargeted metabolomics revealed that live cells were significantly enriched in propionate, indole, and the carotenoid mactraxanthin compared with the supernatant. Propionate is a well‑known activator of PPARγ signaling and fatty acid oxidation,^49^ ^,^ ^50^ and has been reported to reduce hepatic cholesterol accumulation by upregulating CYP7A1-mediated bile acid synthesis and promoting cholesterol clearance.^51^ Whereas indole derivatives can activate aryl hydrocarbon receptor (AhR)-dependent pathways involved in host metabolic regulation.^52^ ^,^ ^53^ In addition, carotenoid-derived retinoic acid has been shown to enhance PPARγ-mediated UCP1 expression and promote thermogenic adipocyte activity.^54^ ^,^ ^55^ ​​These metabolites may therefore contribute to both cholesterol reduction and the activation of BAT thermogenesis observed only in live yeast-treated mice. Consistently, KEGG enrichment analysis of metabolites enriched in viable yeast cells identified pathways related to thermogenesis, adipocyte lipolysis regulation, and oxidative phosphorylation. Collectively, these findings offer a testable metabolic hypothesis for the activation of BAT thermogenesis by live yeast, and explain why the supernatant—despite containing bioactive molecules such as cyclic nucleotides—fails to sustain sufficient concentrations of these labile thermogenic metabolites *in vivo* after a single oral gavage.

Interestingly, although the supernatant failed to induce weight loss or BAT thermogenesis *in vivo*, it exhibited a clear inhibitory effect on lipid accumulation in differentiating 3T3‑L1 preadipocytes, consistent with previous reports on Lactobacillus cell‑free supernatants.^56^ This apparent discrepancy can be explained by fundamental differences between the two settings. In cell culture, the supernatant's bioactive metabolites—including cAMP and cGMP—are present at constant concentrations and have direct access to target cells, allowing sustained signaling that inhibits adipogenic differentiation.^27^ ^,^ ^57-59^ In contrast, following oral administration, these unstable metabolites may undergo dilution, degradation, and rapid clearance, limiting their ability to reach effective concentrations in metabolic tissues. Therefore, although the supernatant exhibits direct anti-adipogenic activity under controlled cellular conditions, sustained metabolic regulation *in vivo* may require continuous production of bioactive compounds by viable colonizing yeast.

In addition to enhanced BAT thermogenesis and metabolic regulation, we observed a significant reduction in cumulative energy intake in mice receiving viable yeast, suggesting that altered feeding behavior may also contribute to the observed anti-obesity phenotype. Given that our findings primarily provide correlational and multi-omics evidence, direct causal mechanisms remain to be fully established. Future research will focus on targeted metabolite interventions (e.g., propionate, indole derivatives, and carotenoid-related compounds) and genetic manipulation to validate their causal roles *in vivo*. Furthermore, extended washout experiments are needed to distinguish stable colonization from continuous input, and the involvement of gut–brain axis pathways—including appetite-regulating hormones such as GLP-1 and PYY—warrants investigation to clarify how viable yeast influences whole-body energy homeostasis and feeding behavior.

## Conclusion

In this study, we first demonstrated that *R. mucilaginosa* DM2025 exhibits favorable safety characteristics and promising probiotic potential. Administration of viable *R. mucilaginosa* DM2025 effectively alleviated high‑fat diet‑induced obesity and was associated with enhanced BAT thermogenic activity, altered metabolic profiles, and improved lipid metabolism. Integrated genomic and metabolomic analyzes suggested that metabolites enriched in viable yeast, including propionate, indole derivatives, and carotenoid-related compounds, may contribute to the activation of the PPARγ–PGC1α–UCP1 thermogenic axis, while direct cholesterol binding to the yeast cell surface provides an additional mechanism for cholesterol reduction. Notably, although the cell-free supernatant reduced serum total cholesterol levels, it failed to induce weight loss or BAT activation, highlighting that cholesterol lowering and systemic anti-obesity effects are distinct physiological outcomes and supporting the concept of viable yeast as a “living factory” capable of continuous metabolic production.

## Supplementary Material

Supplementary Material Supplementary.zip

## Data Availability

The data that support the findings of this study are available on request from the corresponding author [HJL]. The data are not publicly available due to institutional data protection policies.

## ARRIVE guidelines compliance statement

All animal experiments were conducted in accordance with the ARRIVE guidelines (https://arriveguidelines.org). The study design, sample sizes, species/strain (C57BL/6J mice), randomization and blinding procedures, statistical analyzes, and housing/husbandry conditions are described in the Materials and Methods section. All procedures were approved by the Animal Ethics Committee of Dalian Medical University (approval No. XL251114609).
