# Supplementary material for: Rhodotorula mucilaginosa DM2025 ameliorates obesity through cholesterol binding and the gut mycobiome–BAT PPARγ/UCP1 axis
Source: Gut Microbes. 2026 Nov 10;18(1):2760059. doi: 10.1080/19490976.2026.2760059 (PMC13757639; doi:10.1080/19490976.2026.2760059)
Supplement: Supplementary file 1 [file KGMI_A_2760059_SM8565.zip › Supplementary/R_mucilaginosa_supplementary_table.docx]

Table S1. Primers used in the quantitative real-time PCR.

Table S2. Survival rates of *R. mucilaginosa* DM2025 in simulated gastric and intestinal fluids.

Table S3. Survival rates of *R. mucilaginosa* DM2025 after 6 h exposure to bile salt concentrations of 0.1% and 0.3%

Table S1. Primers used in the quantitative real-time PCR

| Gene |  | Sequence (5’-3’) |
| --- | --- | --- |
| PPARγ | PPARγ-F | TTCAAGGGTGCCAGTTTCG |
|  | PPARγ-R | CATCTTTATTCATCAGGGAGGC |
| PGC1α | PGC1α-F | AATGCAGCGGTCTTAGCACT |
|  | PGC1α-R | GTGTGAGGAGGGTCATCGTT |
| UCP1 | UCP1-F | CTTGTTGCCGGGTTTTGCAC |
|  | UCP1-R | ATGGACATCGCACAGCTTGG |
| β‑actin | β‑actin-F | GCCTCACTGTCCACCTTCCA |
|  | β‑actin-R | GCAGCTCAGTAACAGTCCGCCT |

Table S2. Survival of *R. mucilaginosa* DM2025 in simulated gastrointestinal fluids

| Item |  | Simulated intestinal fluid (SIF) |  |  | Simulated gastric fluid (SGF) |  |
| --- | --- | --- | --- | --- | --- | --- |
| exposure time | 0h | 3h | 6h | 0h | 3h | 6h |
| viable cell count (×10⁷ CFU/mL) | 6.1±0.72 | 4.87±0.50 | 3.97±0.35 | 5.3±0.30 | 3.16±0.15 | 2.06±0.10 |
| survival rate（%） |  | 79.87±1.08 | 65.17±2.36 |  | 59.57±0.56 | 38.83±1.17 |

The strain was incubated in simulated gastric fluid (SGF, pH 3.0) and simulated intestinal fluid (SIF, pH 6.8) at 37 °C for up to 6 h. Viable cell counts (×10⁷ CFU/mL) were determined at 0, 3, and 6 h, and survival rates were calculated relative to the initial viable count at 0 h. Data are presented as mean ± SD (n = 3).

| bile salt concentrations (%) | 0.1 | | 0.3 | |
| --- | --- | --- | --- | --- |
| exposure time | 0h | 6h | 0h | 6h |
| Viable cell count (×10⁹ CFU/mL) | 14.23±2.16 | 12.23±3.23 | 11.67±1.53 | 10.33±2.08 |
| survival rate （%） |  | 85±10.69 |  | 88±6.94 |

Table S3. YM liquid medium supplemented with 0.1% and 0.3% bile salts

Viable cell counts were determined at 0 h and 6 h by plate counting, and survival rates were calculated relative to the initial viable count at 0 h (set as 100%). Data are presented as mean ± SD (n = 3).

Table S4. Reagent details

| Reagent | Supplier | Catalogue No. |
| --- | --- | --- |
| YM medium | Prepared in-house | ——— |
| Pepsin | **Beijing Solarbio Science & Technology Co., Ltd.** | P8160 |
| Pancreatin | **Beijing Solarbio Science & Technology Co., Ltd.** | T8150 |
| Bile salt | Shanghai Yuanye Bio-Technology Co., Ltd. | S30895 |
| Cholesterol | **Beijing Solarbio Science & Technology Co., Ltd.** | C8280 |
| TC assay kit | Nanjing Jiancheng Bioengineering Institute | A111-1-1 |
| LDL-C assay kit | Nanjing Jiancheng Bioengineering Institute | A113-1-1 |
| Reverse transcription kit | **Beijing Genstar Biosolutions Co., Ltd.** | A240 |
| SYBR Green Chemistry | **Beijing Genstar Biosolutions Co., Ltd.** | A308 |
| Columbia blood agar plates | **Qingdao Hopebio Biotechnology Co., Ltd.** | HBPM0124-15 |
| Genomic DNA extraction kit | Tiangen Biotech (Beijing) Co., Ltd. | **DP317** |
| 3T3-L1 preadipocytes | Suzhou Haixing Biotechnology Co., Ltd. | ——— |
