# Supplementary material for: Rhodotorula mucilaginosa DM2025 ameliorates obesity through cholesterol binding and the gut mycobiome–BAT PPARγ/UCP1 axis
Source: Gut Microbes. 2026 Nov 10;18(1):2760059. doi: 10.1080/19490976.2026.2760059 (PMC13757639; doi:10.1080/19490976.2026.2760059)
Supplement: Supplementary file 1 [file KGMI_A_2760059_SM8565.zip › Supplementary/Supplement Figure captions.docx]

**Supplement Figure**

**Supplementary Fig S1. Auto-aggregation of *R. mucilaginosa* DM2025.** The strain was incubated at 37 °C without agitation for 12 h. Auto-aggregation percentage was calculated as 1 − (A₁/A₀) × 100%, where A₀ is the initial absorbance at 620 nm and A₁ is the absorbance at each indicated time point.

**Supplementary Fig S2. Genomic safety assessment of *R. mucilaginosa* DM2025.** (A) Classification statistics of virulence factor homologs against the DFVF (Virulence Factors Database); (B) Annotation results of antimicrobial resistance genes against the CARD (Comprehensive Antibiotic Resistance Database); (C) Annotation results of pathogenicity-related genes against the PHI (Pathogen–Host Interaction Database).

**Supplementary Fig S3. Bacterial community composition in cecal contents of mice.** (A) Relative abundance of bacterial taxa at the phylum level. (B) Relative abundance of bacterial taxa at the genus level. Data were obtained from metagenomic sequencing of cecal samples (n = 5 per group).

**Supplementary Fig S4. Microbial dysbiosis index (MDI) at the ASV level.** (A) MDI was significantly higher in the HFD group compared with the NC group. (B) MDI was significantly reduced in the HFD+RM group compared with the HFD group. Data are shown as mean ± SD (n = 5). (**P*<0.05, ***P*<0.01, ****P* < 0.001).

**Supplementary Fig S5. GO enrichment analysis of lipid metabolism-related genes in the *R. mucilaginosa* DM2025 genome.** Terms are grouped into biological process, cellular component, and molecular function. Dot size indicates gene count; dot color indicates the adjusted P value.

**Supplementary Fig S6. KEGG enrichment analysis of lipid metabolism-related pathways in the *R. mucilaginosa* DM2025 genome.** The x-axis shows the rich factor. Dot size indicates gene count; dot color indicates the adjusted P value.

**Supplementary Fig S7. Weekly energy intake in Experiments 1 and 2.** (A) Experiment 1: NFD+NS, HFD+NS, HFD+RM (n = 7 per group). (B) Experiment 2: NFD+NS, HFD+NS, HFD+RM, HFD+SUP (n = 8 per group). (**P*<0.05, ***P*<0.01, ****P* < 0.001)).
